# Livestock Animal Hair as an Indicator of Environmental Heavy Metals Pollution in Central Albania

**DOI:** 10.3390/ani15131898

**Published:** 2025-06-27

**Authors:** Marta Castrica, Egon Andoni, Alda Quattrone, Xhelil Koleci, Enkeleda Ozuni, Pellumb Zalla, Rezart Postoli, Laura Menchetti, Bengü Bilgiç, Duygu Tarhan, Ibrahim Ertugrul Yalcin, Ilir Dova, Nour Elhouda Fehri, Mehmet Erman Or, Albana Munga, Doriana Beqiraj, Giulio Curone, Stella Agradi

**Affiliations:** 1 Department of Comparative Biomedicine and Food Science, University of Padova, Agripolis, Viale dell’Università 16, 35020 Legnaro, Italy; marta.castrica@unipd.it; 2 Department of Public Health, Veterinary Faculty of Tirana, Agricultural University of Tirana, Rr Pajsi Vodica, Koder-Kamez, 1029 Tirane, Albania; eandoni@ubt.edu.al (E.A.); xhelil.koleci@ubt.edu.al (X.K.); enkeleda.ozuni@ubt.edu.al (E.O.); pellumb.zalla@ubt.edu.al (P.Z.); rezart.postoli@ubt.edu.al (R.P.); idova@ubt.edu.al (I.D.); amunga@ubt.edu.al (A.M.); dbeqiraj@ubt.edu.al (D.B.); 3 Department of Veterinary Medicine and Animal Sciences (DIVAS), University of Milan, Via dell’Università 6, 26900 Lodi, Italy; alda.quattrone@unimi.it (A.Q.); nour.fehri@unimi.it (N.E.F.); giulio.curone@unimi.it (G.C.); 4 School of Biosciences and Veterinary Medicine, University of Camerino, Via Circonvallazione 93/95, 62024 Matelica, Italy; 5 Department of Internal Medicine, Faculty of Veterinary Medicine, Istanbul University-Cerrahpasa, Avcilar, Istanbul 34320, Türkiye; bengu.bilgic@iuc.edu.tr (B.B.); ermanor@iuc.edu.tr (M.E.O.); 6 Department of Biophysics, School of Medicine, Bahcesehir University, Istanbul 34734, Türkiye; duygu.tarhan@bau.edu.tr; 7 Department of Civil Engineering, Faculty of Engineering and Natural Sciences, Bahcesehir University, Istanbul 34353, Türkiye; ibrahimertugrul.yalcin@bau.edu.tr; 8 Department of Veterinary Medicine, University of Torino, Largo Paolo Braccini, 2, 10095 Grugliasco, Italy; stella.agradi@unito.it

**Keywords:** hair, environment, survey, Albania, bioindicator

## Abstract

Trace elements, which may have harmful health effects, are present in the environment at varying concentrations. In Albania, data on exposure risks are limited. This study aimed to assess and compare the concentrations of various trace elements (aluminum, arsenic, boron, calcium, cadmium, chromium, copper, iron, potassium, magnesium, manganese, nickel, lead, and zinc) in the hair of cattle and sheep raised in Central Albania (Tirana and Elbasan Counties). Hair samples were collected from 25 cattle and 25 sheep per county and analyzed using inductively coupled plasma–optical emission spectroscopy. Zinc concentrations were significantly higher in cattle than in sheep (*p* = 0.029), while no differences were observed between counties (*p* > 0.05), indicating similar environmental conditions. Copper (17.84, 95%CI: 13.63–16.34 and 15.84, 95%CI: 14.00–17.69 mg/kg in cattle, and 15.58, 95%CI: 13.61–17.56 and 14.14, 95%CI: 12.07–16.20 mg/kg in sheep, in Elbasan and Tirana County, respectively), arsenic (2.08, 95%CI: 1.45–1.21 and 1.51, 95%CI: 1.19–1.81 mg/kg in cattle, 1.73, 95%CI: 1.38–2.07 and 1.39, 95%CI: 1.02–1.75 mg/kg in sheep, in Elbasan and Tirana County, respectively), and cadmium (2.36, 95%CI: 1.63–2.07 and 2.00, 95%CI: 1.68–2.32 mg/kg in cattle, 2.00, 95%CI: 1.59–2.40 and 1.71, 95%CI: 1.39–2.02 mg/kg in sheep, in Elbasan and Tirana County, respectively) concentrations exceeded the values reported in the literature, likely due to contamination from local mining and metal processing activities. Further research is needed to determine the sources of contamination and assess potential risks to animal and human health.

## Simple Summary

Trace elements are substances of natural or anthropogenic origin present in the environment that may pose health risks to humans. Domestic herbivores are continuously exposed to these elements through environmental contact. In Albania, the geological features and industrial or metallurgical activities in certain areas may increase the likelihood of exposure to specific trace elements. This study aimed to evaluate and compare the concentrations of various trace elements (aluminum, arsenic, boron, calcium, cadmium, chromium, copper, iron, potassium, magnesium, manganese, nickel, lead, and zinc) in the hair of cattle and sheep raised in two counties of Central Albania (Tirana and Elbasan). Fifty healthy female animals (i.e., twenty-five bovines and twenty-five sheep) from each county were enrolled, and a hair sample was collected and analyzed. No significant differences in trace element concentrations were found between the two counties. However, zinc levels were significantly higher in cattle than in sheep. Compared to values reported in the literature, concentrations of iron, nickel, chromium, and copper were elevated, likely due to the mineral-rich soil in Central Albania. Additionally, arsenic and cadmium levels were comparable to those found in highly polluted areas, probably as a result of local mining and metal foundry activities. In conclusion, hair analysis in domestic herbivores represents a reliable and non-invasive method for biomonitoring environmental contamination by trace elements.

## 1. Introduction

Essential and non-essential elements, collectively referred to as trace elements, are classified according to their physiological roles and occur naturally in the environment [1]. Among them, heavy metals are a subgroup of trace elements that, like others, originate from both natural sources (e.g., soil erosion, weathering of the Earth’s crust) and anthropogenic activities (e.g., industrial effluents, urban runoff, sewage discharge, insecticides, and mining) [2]. The accumulation of these elements is closely linked to environmental pollution, particularly in areas identified as contamination hotspots. Once released into the environment, heavy metals and other trace elements are non-degradable and can enter animals primarily through soil, water, the food chain, or inhalation. Chronic exposure to heavy metals through multiple pathways can lead to their progressive accumulation in various body tissues [3]. Due to their chemical properties, heavy metals can bypass cellular control mechanisms and bind to native proteins, DNA, and nuclear proteins, disrupting biological functions and causing toxicity and oxidative damage to internal organs [2]. These heavy metals exhibit mutagenic, teratogenic, and carcinogenic effects, and even at low doses they can impair body condition, reduce reproductive performance, and suppress the immune system in domestic animals [4,5]. The severity of heavy metal toxicity depends on the animal’s metabolic status related to trace elements, as these metals can also disrupt normal trace element metabolism [6]. Among them, mercury, cadmium, and lead are particularly toxic, posing significant risks even at minimal exposure levels [6]. Thus, the exposure to excessive concentrations of heavy metals in the environment can seriously threaten the health and welfare of both animals and humans [7], owing to their well-documented harmful effects on living organisms [8].

To mitigate their toxic impact, the body employs various biochemical mechanisms to sequester heavy metals and other trace elements. Hair serves as an important biological matrix for bioaccumulation, providing valuable information on long-term exposure [9]. Indeed, due to its high keratin content, hair can bind divalent cations (e.g., lead and cadmium) through its sulfhydryl groups, allowing these heavy metals to accumulate in this biological matrix for months [8]. In fact, hair also provides valuable information on the metabolic pools of trace elements in animals [10]. Unlike urine and blood, which reflect short-term exposure, hair analysis enables the assessment of long-term exposure, offering insights into changes over time [11]. Among domestic and wild animals, herbivores are continuously exposed to trace elements uptake through ingestion of small amounts of soil, contaminated vegetation, and water [12]. Multiple studies have shown that herbivore hair accurately reflects the accumulation and concentration of trace elements in the surrounding environment [12,13,14]. Moreover, hair sampling is a non-invasive and efficient screening method, as it can be easily collected from various species without requiring special preparation. However, differences in trace element accumulation in the hair of domestic herbivores (e.g., cattle, sheep, goat, camel) remain poorly studied.

Large areas of soil in Albania originate from serpentine rocks, a geological formation known to contain significant reserves of iron, nickel, chromium, and copper. Combined with the presence of mines and metal foundries in the region [15], this poses a potential risk of heavy metal exposure for both animals and humans. Although several studies have focused on environmental monitoring of heavy metals in Albania, particularly in aquatic environments and through risk assessments related to fish consumption [16,17,18], there are currently no data on the bioaccumulation of these elements in domestic animals raised for human consumption. Such information is critical for evaluating potential public health risks. Given Albania’s ongoing efforts to align with the EU *acquis* concerning environmental management, food safety, and veterinary policy, further research in these areas is essential [19].

The study hypothesizes that trace elements can be detected in animal hair and that their concentrations are influenced primarily by the region where the animals live, rather than by the species, assuming that domestic herbivores share similar feeding behaviors, as in the case of bovines and sheep. Therefore, the objective of this study was to evaluate and compare the levels of various trace elements, including heavy metals (aluminum (Al), arsenic (As), boron (B), calcium (Ca), cadmium (Cd), chromium (Cr), copper (Cu), iron (Fe), potassium (K), magnesium (Mg), manganese (Mn), nickel (Ni), lead (Pb), and zinc (Zn)) in the hair of cows and sheep farmed in the two counties of Central Albania (Tirana and Elbasan).

## 2. Materials and Methods

### 2.1. Study Area and Animals

**Figure 1:**
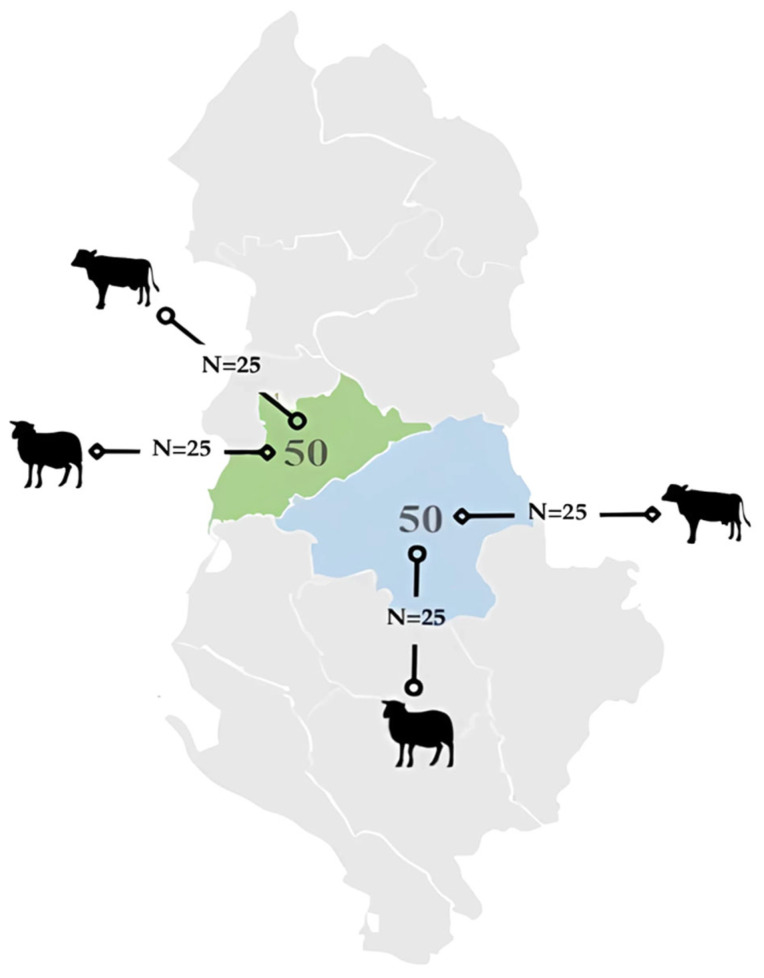
Total number of animals (n = 100) and number of animals per species involved in the study (green: Tirana County; blue: Elbasan).

Central Albania consists of two counties, Tirana and Elbasan, which share similar geological features, including large soil reserves of iron, nickel, chromium, and copper, as well as the presence of mines and metal foundries. For this reason, the animals enrolled in the study were domestic herbivores (i.e., cows and sheep) raised in these two Counties. A total of 100 animals were enrolled, namely 50 from Tirana County (25 cows and 25 sheep) and 50 from Elbasan County (25 cows and 25 sheep) (Figure 1).

All animals enrolled in the study were lactating and were kept in a semi-extensive farming system, with free grazing on pasture during the day and indoor housing with locally produced hay feeding during the dark hours. The subjects were randomly selected from a population of 129 cows and 1356 sheep healthy adult females (mean age ± SD: 3.76 ± 1.06 and 3.86 ± 1.05, for cows and sheep, respectively) across 100 farms located in both Tirana and Elbasan counties. The study was conducted from April to August 2024. Hair samples were obtained from the left rump of each animal by clipping an area of approximately 10 cm^2^ as close to the skin as possible using an electric clipper. The samples were stored in glass containers at room temperature and were protected from sunlight until analysis. Hair collection was performed on live animals in compliance with current animal welfare legislation, and only clinically healthy individuals were included in the study.

### 2.2. Hair Collection and Analysis

For each animal enrolled in the study, a hair sample was collected from the left abdominal region, just behind the costal arch, from an area measuring 10 × 10 cm^2^, using scissors disinfected with ethyl alcohol. Each sample was then sealed in a polyethylene bag and stored in a light-protected area at room temperature until analysis.

For the analysis, between 0.2000 and 0.2500 g of hair from each sample was weighed using an analytical balance and transferred into Teflon vessels of a microwave digestion system (Berghof MWS2). Each sample was then treated with 8 mL of 65% HNO_3_, Merck (Darmstadt, Germany). The microwave digestion system conducted the mineralization process following the 145 °C temperature and 20 min time protocol. After digestion, the samples were filtered through Whatman blue band filter paper and transferred into sterile 50 mL Falcon^®^ tubes (Corning, Corning, NY, USA), using ultrapure water to adjust the final volume to 50 mL. Subsequently, the concentrations of aluminum (Al), arsenic (As), boron (B), calcium (Ca), cadmium (Cd), chromium (Cr), copper (Cu), iron (Fe), potassium (K), magnesium (Mg), manganese (Mn), nickel (Ni), lead (Pb), and zinc (Zn) were determined using inductively coupled plasma–optical emission spectroscopy (ICP-OES, PerkinElmer-Optima 7000DV; PerkinElmer Inc., Waltham, MA, USA) with multi-element standard solutions (1000 mg L^−1^) Merck (Darmstadt, Germany). The plasma gas flow rate was set to 17 L/min, while the argon carrier flow rate was maintained at 0.2 L/min. The sample flow rate was adjusted to 1.50 L/min. A flush time of 15 s was applied between samples to prevent cross-contamination. The radio frequency (RF) power was set at 1450 W during analysis.

The multi-element standard solutions were prepared at the 10–25–50–100–250–500–1000 ppb (mg/kg) concentrations, respectively. The calibration curve was obtained from the ICP-OES analyzer using blank and multi-element standard solutions. The concentration of each element was quantified individually, with consideration given to the weight of the respective sample. The results of the ICP-OES method validation parameters, LoD (limit of detection), and LoQ (limit of quantification) are given in Table 1.

Quality control was carried out at three concentration levels using standard solutions and blanks as reference materials. The recovery rates for all analyzed elements ranged between 99.9% and 100.1%, indicating the method’s high accuracy and reliability (Table 2).

### 2.3. Statistical Analysis

Descriptive statistics were used to present data as means, standard deviations (SD), medians (Md), minimums (Min), and maximums (Max). Diagnostic graphs and Shapiro–Wilk tests were used to verify the normality. All variables were log-transformed to improve their distribution, but raw data are presented in the figures and tables. The values recorded in Elbasan and Tirana, and in bovines and sheep (regardless of the county), were compared using independent *t*-tests. Levene’s test was used to verify the equality of variance, and Welch’s correction was used when this assumption was not met.

Finally, regardless of species and collection site, the associations between elements were evaluated using Spearman’s rho coefficient (ρ). The correlation was considered weak if ρ < |0.39|, moderate if ρ = 0.40–0.69, strong if ρ = 0.70–0.89, and very strong if ρ > 0.89 [20].

Statistical analyses were performed with SPSS 25.0 (SPSS Inc., Chicago, IL, USA). A *p*-value < 0.05 was considered significant, while a *p*-value between 0.05 and 0.1 was considered a trend. Furthermore, graphs were generated using GraphPad software Version 8.0 (GraphPad, San Diego, CA, USA). Graphical representations were limited to elements with statistically significant interspecies differences (bovines vs. sheep).

## 3. Results

Mean, standard deviation, median, minimum, and maximum concentrations of heavy metals levels for sheep and bovines are given in Table 3 and Table 4, respectively. No metal showed significant differences between the Tirana and Elbasan Counties. A trend was found only for As and Mn in sheep, both of which were higher in Elbasan than in Tirana (*p* < 0.1).

**Figure 2:**
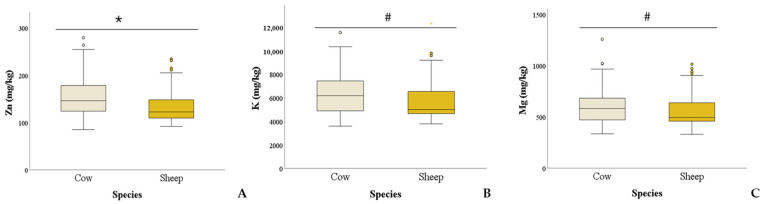
Box plots comparing Zn (Panel **A**), K (Panel **B**), and Mg (Panel **C**) concentrations in bovines and sheep. * *p* < 0.05, ^#^ *p* < 0.1.

Regarding species differences, Zn was higher in bovines than sheep (*p* = 0.029). A trend was also found for K (*p* = 0.079) and Mn (*p* = 0.085), which were higher in bovines than in sheep (Figure 2).

Table 5 shows the correlation coefficients between elements. The strongest correlations (ρ > 0.9) were found between Al and As, As and Cu, As and Fe, Cu and Fe, and Cu and Ni (*p* < 0.01).

## 4. Discussion

Given that animal hair is a reliable bioindicator of environmental contamination by trace elements, the objective of this study was to assess contamination levels in the hair of cows and sheep raised in Central Albania, and to investigate potential differences between the two species and between the two counties considered (Tirana and Elbasan). Regarding interspecies differences, only zinc levels were significantly higher in cows’ hair (*p* = 0.029) compared to in sheep. While the literature generally reports comparable zinc concentrations in the hair of both species (i.e., 106.3 ± 7.4 mg/kg in cattle [21] and 95.3 ± 21.2 mg/kg in sheep from unpolluted areas [22]), and considering their similar foraging behavior [23], the observed difference is likely due to variations in environmental contamination at specific grazing sites. This may have occurred despite efforts to ensure spatial homogeneity among the sampled animals.

On the other hand, no significant differences were observed between Tirana and Elbasan, suggesting that the environmental contamination by the trace elements studied is homogeneous across the two counties. This result was particularly expected, given the significant geological similarities between the two areas in terms of soil composition [16]. Additionally, from an anthropogenic contamination perspective, Tirana and Elbasan are among the four most populated counties in Albania [24]. More importantly, they represent the country’s most developed region, hosting over 40 percent of the national companies [25].

The results obtained are particularly interesting, especially when considering the average levels of trace elements detected in the hair of the examined animals. As expected, the concentrations of iron, nickel, chromium, and copper were notably high, likely reflecting the region’s geological composition. In particular, copper levels (17.84, 95%CI: 13.63–16.34 and 15.84, 95%CI: 14.00–17.69 mg/kg in cows, and 15.58, 95%CI: 13.61–17.56 and 14.14, 95%CI: 12.07–16.20 mg/kg in sheep, in Elbasan and Tirana County, respectively) were significantly higher than those reported in the literature, even surpassing values found in cattle grazing near a closed lead-cum-operational zinc smelter (11.51 ± 2.17 mg/kg) [21]. The elevated copper concentrations may not be solely attributed to emissions from metallurgical industries but also to the widespread agricultural use of copper sulphate as a fungicide. This practice has been debated for years in the EU and beyond due to copper sulphate’s tendency to accumulate in the soil [26]. However, none of the animals in our study showed clinical signs of copper intoxication, despite belonging to species that are generally more susceptible to copper poisoning due to their lower efficiency in terms of copper excretion [27]. This finding suggests that the detected copper levels were insufficient to induce oxidative damage. The concentrations of iron, nickel, and chromium detected in the hair of animals from Central Albania were consistent with values reported in the scientific literature. Specifically, the iron levels were 463, 95%CI: 375–446 and 437, 95%CI: 381–492 mg/kg in cows, and 432, 95%CI: 382–480 and 384, 95%CI: 337–432 mg/kg in sheep, in Elbasan and Tirana Counties, respectively. The nickel levels were 3.42, 95%CI: 2.51–3.11 and 2.96, 95%CI: 2.55–3.36 mg/kg in cows, and 3.01,95%CI: 2.47–3.54 and 2.67, 95%CI: 2.20–3.13 mg/kg in sheep. Chromium concentrations were 5.58, 95%CI: 3.87–4.89 and 4.53, 95%CI: 3.87–5.18 mg/kg in cows, and 4.71, 95%CI: 3.89–5.52 and 4.23, 95%CI: 3.42–5.05 mg/kg in sheep. These values align with the literature data for cattle (175.13 ± 196.38 mg/kg for iron, 3.73 ± 4.34 mg/kg for nickel, and 5.66 ± 6.68 mg/kg for chromium [28]) and for sheep (with values of 128 ± 19 till 996 ± 121 mg/kg for iron, 0.60 ± 0.17 till 1.75 ± 0.65 mg/kg for nickel, depending on environmental pollution levels [13]). Similarly, lead and zinc levels remained within physiological ranges (i.e., 106.3 ± 7.4 mg/kg in cows [21] and 95.3 ± 21.2 mg/kg in sheep [22] for zinc; 3.00 ± 0.44 [21] or 2.99 ± 0.27 mg/kg [8] in cows and 2 ± 0.21 mg/kg in sheep [13] for lead), with lower values than those observed in animals raised in contaminated industrial areas (i.e., 509.1 ± 127.2 mg/kg in cattle [21] for zinc; 36.40 ± 7.67 [21] or 15.09 ± 0.85 mg/kg [8] in cattle and 6 ± 0.72 mg/kg in sheep [13] for lead) [8,13,21,22]. In contrast, arsenic concentrations (2.08, 95%CI: 1.45–1.21 and 1.51, 95%CI: 1.19–1.81 mg/kg in cattle, 1.73, 95%CI: 1.38–2.07 and 1.39, 95%CI: 1.02–1.75 mg/kg in sheep, in Elbasan and Tirana County, respectively) were notably high compared to previously reported values (i.e., 0.43 ± 0.05 [29] or 0.25 mg/kg [30] in cattle and 0.53 ± 0.04 [31] or 0.07 [32] mg/kg in sheep). This finding is particularly significant given arsenic’s high toxicity, even at low doses. Arsenic is naturally present in rocks and sediments, but environmental contamination is often linked to human industrial activities [33,34]. However, recent analyses (2022) indicate that the mean arsenic concentrations in Albania’s agricultural and forest soils are below the global average of 12 mg/kg [16]. This suggests that the elevated arsenic levels detected in hair samples may be due to recent anthropogenic contamination.

As for cadmium, a heavy metal with no detectable beneficial biological function, the concentrations observed in this study are comparable to those found in highly polluted regions, such as areas near active smelters (i.e., 2.62 ± 0.32 mg/kg [8]) or closed lead-cum-operational zinc smelters (i.e., 16.44 ± 5.49 mg/kg [21]). Given the presence of mines and particularly metal foundries in the studied area [15], the elevated cadmium concentrations in hair samples can likely be attributed to human industrial activities. These industries may still be insufficiently regulated or lacking adequate measures to prevent the release of waste products into the environment. Finally, aluminum is another element for which the values found in this study were higher than those reported in the literature (i.e., 61.34 ± 71.86 mg/kg) [28]. However, aluminum concentrations in the hair of domestic ruminants remain poorly studied. Consequently, it is currently unclear whether the values observed in our study indicate meaningful environmental contamination that could pose a risk to animal or human health.

## 5. Conclusions

In conclusion, analyzing the hair of domestic herbivores offers a simple yet effective method for monitoring environmental contamination by trace elements. The choice of species, whether cows or sheep, appears to have minimal influence, as only zinc levels showed a significant difference between the two in our study. While geological surveys of soil composition are essential for assessing the risk of heavy metal exposure, hair analysis serves as an equally valuable bioindicator. It not only reflects soil contamination but also provides insight into anthropogenic factors, such as the presence of smelters, mines, industrial activities, and high-traffic roads, which contribute to the contamination of soil, water, and vegetation. Notably, this study revealed particularly high concentrations of arsenic and cadmium, which do not align with the region’s known geological profile. This suggests that recent human activities may be responsible. Further research is needed to identify the sources of these contaminants and to evaluate the potential risks they pose to both animal and human health.

## Figures and Tables

**Table 1:** Parameters of the analytical method for ICP-OES.

Element	Wavelength (nm)	LoD (mg·kg^−1^)	LoQ (mg·kg^−1^)	RSD (%)	R^2^
**Al**	396.153	0.644	2.147	0.93	0.999891
**As**	188.979	0.007	0.023	0.85	0.999902
**B**	249.677	0.401	1.337	0.99	0.999915
**Ca**	317.933	0.894	2.980	0.94	0.999896
**Cd**	228.802	0.005	0.017	0.87	0.999919
**Cr**	267.716	0.009	0.030	0.97	0.999873
**Cu**	327.393	0.095	0.317	1.01	0.999924
**Fe**	238.204	0.341	1.137	1.12	0.999849
**K**	766.490	1.358	4.527	1.24	0.999899
**Mg**	285.213	0.512	1.707	0.88	0.999928
**Mn**	257.610	0.099	0.330	1.05	0.999812
**Ni**	231.604	0.013	0.043	0.91	0.999894
**Pb**	220.353	0.011	0.037	0.89	0.999884
**Zn**	213.857	0.152	0.507	1.11	0.999918

LoD: limit of detection; LoQ: limit of quantification; RSD: relative standard deviation; R^2^: determination coefficient.

**Table 2:** The quality control parameters of all elements for the ICP-OES method.

Element	Quality Control (QC)	Expected Concentration (ppb)	Measured Concentration (n = 3) (ppb)	Recovery (%)
**Al, As, B, Ca, Cd, Cr, Cu, Fe, K, Mg, Mn, Ni, Pb, Zn**	QC-1 QC-2 QC-3	25 250 1000	25 251 999	100 100.4 99.9

QC: quality control; ppb: mg·kg^−1^; Al: aluminum; As: arsenic; B: boron; Ca: calcium; Cd: cadmium; Cr: chromium; Cu: copper; Fe: iron; K: potassium; Mg: magnesium; Mn: manganese; Ni: nickel; Pb: lead; Zn: zinc.

**Table 3:** Mean with 95% confidence interval (CI), standard deviation (SD), median (Md), minimum (Min), and maximum (Max) concentrations of heavy metals and trace elements in sheep in two different counties, expressed in mg·kg^−1^, and *p*-value of the comparison between counties.

Elements	Parameter	County		*p* Value *
		Elbasan	Tirana	
Al	Mean ± SD 95%CI for mean Md Min–Max	151 ± 50 130–172 136 90–313	140 ± 38 124–156 137 85–224	0.449
As	Mean ± SD 95%CI for mean Md Min–Max	1.73 ± 0.84 1.38–2.07 1.55 0.46–4.03	1.39 ± 0.89 1.02–1.75 1.22 0.46–3.43	0.084
B	Mean ± SD 95%CI for mean Md Min–Max	1067 ± 288 947–1186 978 684–1720	1048 ± 377 893–1203 916 636–2045	0.659
Ca	Mean ± SD 95%CI for mean Md Min–Max	2605 ± 822 2266–2944 2267 1615–4736	2380 ± 838 2034–2726 2248 1408–4507	0.262
Cd	Mean ± SD 95%CI for mean Md Min–Max	2.00 ± 0.98 1.59–2.40 1.82 0.61–4.37	1.71 ± 0.76 1.39–2.02 1.58 0.68–3.75	0.330
Cr	Mean ± SD 95%CI for mean Md Min–Max	4.71 ± 1.97 3.89–5.52 4.24 2.39–11.77	4.23 ± 1.97 3.42–5.05 3.62 2.51–10.01	0.276
Cu	Mean ± SD 95%CI for mean Md Min–Max	15.58 ± 4.78 13.61–17.56 14.06 9.67–27.31	14.14 ± 5.00 12.07–16.20 12.69 9.79–27.13	0.209
Fe	Mean ± SD 95%CI for mean Md Min–Max	432 ± 119 382–480 404.14 266–802	384 ± 115 337–432 370 227–634	0.124
K	Mean ± SD 95%CI for mean Md Min–Max	6107 ± 1966 5295–6918 5434 3846–12329	5489 ± 1633 4815–6163 4878 3798–9803	0.204
Mg	Mean ± SD 95%CI for mean Md Min–Max	585 ± 153 521–648 521 367–1016	538 ± 188 460–615 473 331–972	0.190
Mn	Mean ± SD 95%CI for mean Md Min–Max	5.63 ± 2.03 4.79–6.47 4.92 2.96–12.38	4.83 ± 1.82 4.08–5.58 4.27 2.80–9.58	0.097
Ni	Mean ± SD 95%CI for mean Md Min–Max	3.01 ± 1.30 2.47–3.54 2.46 1.33–6.58	2.67 ± 1.13 2.20–3.13 2.24 1.33–5.20	0.300
Pb	Mean ± SD 95%CI for mean Md Min–Max	2.63 ± 1.08 2.18–3.08 2.39 1.13–6.00	2.27 ± 1.18 1.78–2.76 2.11 0.97–4.80	0.130
Zn	Mean ± SD 95%CI for mean Md Min–Max	140 ± 38 124–155 134 92–235	135 ± 41 118–151 122 93–232	0.562

* After log-transformation.

**Table 4:** Mean with 95% confidence interval (CI), standard deviation (SD), median (Md), minimum (Min), and maximum (Max) concentrations of heavy metals and trace elements in bovines in two different counties, expressed in mg·kg^−1^, and *p*-value of the comparison between areas.

**Elements**	**Parameter**	**County**		***p* Value** *
		**Elbasan**	**Tirana**	
Al	Mean ± SD 95%CI for mean Md Min–Max	165 ± 64 132–159 150 93–307	151 ± 53 129–173 139 95–321	0.461
As	Mean ± SD 95%CI for mean Md Min–Max	2.08 ± 1.41 1.45–1.21 1.67 0.54–5.80	1.51 ± 0.76 1.19–1.81 1.28 0.596–3.37	0.219
B	Mean ± SD 95%CI for mean Md Min–Max	1235 ± 453 972–1152 1241 647–2121	1075 ± 252 971–1179 1035 673–1601	0.252
Ca	Mean ± SD 95%CI for mean Md Min–Max	2764 ± 921 2249–2689 2916 1569–4613	2557 ± 708 2264–2850 2591 1438–3993	0.458
Cd	Mean ± SD 95%CI for mean Md Min–Max	2.36 ± 1.37 1.63–2.07 2.27 0.72–5.88	2.00 ± 0.79 1.68–2.32 2.00 0.78–4.05	0.585
Cr	Mean ± SD 95%CI for mean Md Min–Max	5.58 ± 2.74 3.87–4.89 5.19 2.43–12.06	4.53 ± 1.59 3.87–5.18 4.49 2.44–7.73	0.179
Cu	Mean ± SD 95%CI for mean Md Min–Max	17.84 ± 7.75 13.63–16.34 17.08 9.07–36.53	15.84 ± 4.48 14.00–17.69 14.93 9.76–25.99	0.489
Fe	Mean ± SD 95%CI for mean Md Min–Max	463 ± 168 375–446 461 241–810	437 ± 133 381–492 400 258–752	0.681
K	Mean ± SD 95%CI for mean Md Min–Max	6746 ± 2358 5373–6287 6638 3603–11566	6171 ± 1537 5536–6805 6094 3883–9813	0.497
Mg	Mean ± SD 95%CI for mean Md Min–Max	653 ± 246 509–597 660 365–1258	567 ± 114 520–614 579 336–836	0.282
Mn	Mean ± SD 95%CI for mean Md Min–Max	6.20 ± 2.72 4.75–5.72 5.98 2.77–12.52	5.63 ± 1.52 5.00–6.26 5.30 3.30–9.69	0.667
Ni	Mean ± SD 95%CI for mean Md Min–Max	3.42 ± 1.73 2.51–3.11 3.19 1.31–7.47	2.96 ± 0.98 2.55–3.36 2.96 1.51–5.64	0.543
Pb	Mean ± SD 95%CI for mean Md Min–Max	3.02 ± 1.63 2.07–2.70 3.18 0.93–6.94	2.50 ± 1.03 2.08–2.93 2.30 1.15–4.83	0.417
Zn	Mean ± SD 95%CI for mean Md Min–Max	162 ± 57 131–153 151 85–280	150 ± 35 135–164 142 103–232	0.571

* After log-transformation.

**Table 5:** Correlations (Spearman’s rho) between metals independent of species and sampling site.

	Al	As	B	Ca	Cd	Cr	Cu	Fe	K	Mg	Mn	Ni	Pb
**As**	0.900	--											
**B**	0.869	0.890	--										
**Ca**	0.824	0.874	0.844	--									
**Cd**	0.793	0.774	0.829	0.716	--								
**Cr**	0.835	0.897	0.888	0.882	0.799	--							
**Cu**	0.870	0.930	0.894	0.866	0.774	0.873	--						
**Fe**	0.864	0.910	0.877	0.837	0.793	0.895	0.902	--					
**K**	0.829	0.868	0.877	0.810	0.838	0.896	0.871	0.872	--				
**Mg**	0.790	0.882	0.846	0.824	0.765	0.861	0.876	0.872	0.873	--			
**Mn**	0.814	0.870	0.878	0.832	0.834	0.880	0.857	0.884	0.882	0.888	--		
**Ni**	0.838	0.864	0.890	0.800	0.836	0.805	0.911	0.844	0.859	0.833	0.870	--	
**Pb**	0.815	0.861	0.847	0.845	0.814	0.868	0.854	0.863	0.836	0.819	0.840	0.808	--
**Zn**	0.781	0.815	0.845	0.743	0.784	0.793	0.861	0.860	0.840	0.818	0.870	0.893	0.773

Correlation is significant at the 0.01 level (2-tailed) for all.

## Data Availability

The raw data supporting the conclusions of this article will be made available by the authors on request.
